# *N*-Phenethyl Substitution in 14-Methoxy-*N*-methylmorphinan-6-ones Turns Selective µ Opioid Receptor Ligands into Dual µ/δ Opioid Receptor Agonists

**DOI:** 10.1038/s41598-020-62530-w

**Published:** 2020-03-27

**Authors:** Maria Dumitrascuta, Marcel Bermudez, Tanila Ben Haddou, Elena Guerrieri, Lea Schläfer, Andreas Ritsch, Sandor Hosztafi, Aquilino Lantero, Christoph Kreutz, Dominique Massotte, Helmut Schmidhammer, Gerhard Wolber, Mariana Spetea

**Affiliations:** 1 Department of Pharmaceutical Chemistry, Institute of Pharmacy and Center for Molecular Biosciences Innsbruck (CMBI), University of Innsbruck, 6020 Innsbruck, Austria; 2 Institute of Pharmacy, Freie Universität Berlin, D-14195 Berlin, Germany; 3 Department of Pharmaceutical Chemistry, Semmelweis University, H-1092 Budapest, Hungary; 4 Institute of Organic Chemistry, Center for Molecular Biosciences Innsbruck (CMBI), University of Innsbruck, 6020 Innsbruck, Austria; 5 Centre de la Recherche Nationale Scientifique, Université de Strasbourg, Institut des Neurosciences Cellulaires et Intégratives, 67000 Strasbourg, France

**Keywords:** Drug discovery, Medicinal chemistry, Pharmacology, Preclinical research, Drug development, G protein-coupled receptors, Molecular modelling, Cheminformatics, Medicinal chemistry

## Abstract

Morphine and structurally-derived compounds are µ opioid receptor (µOR) agonists, and the most effective analgesic drugs. However, their usefulness is limited by serious side effects, including dependence and abuse potential. The N-substituent in morphinans plays an important role in opioid activities *in vitro* and *in vivo*. This study presents the synthesis and pharmacological evaluation of new *N*-phenethyl substituted 14-*O*-methylmorphinan-6-ones. Whereas substitution of the *N*-methyl substituent in morphine (**1**) and oxymorphone (**2**) by an *N*-phenethyl group enhances binding affinity, selectivity and agonist potency at the µOR of **1a** and **2a**, the *N-*phenethyl substitution in 14-methoxy-*N*-methylmorphinan-6-ones (**3** and **4**) converts selective µOR ligands into dual µ/δOR agonists (**3a** and **4a**). Contrary to *N*-methylmorphinans **1**–**4**, the *N*-phenethyl substituted morphinans **1a**–**4a** produce effective and potent antinociception without motor impairment in mice. Using docking and molecular dynamics simulations with the µOR, we establish that *N*-methylmorphinans **1–4** and their *N*-phenethyl counterparts **1a**–**4a** share several essential receptor-ligand interactions, but also interaction pattern differences related to specific structural features, thus providing a structural basis for their pharmacological profiles. The emerged structure-activity relationships in this class of morphinans provide important information for tuning *in vitro* and *in vivo* opioid activities towards discovery of effective and safer analgesics.

## Introduction

**Figure 1:**
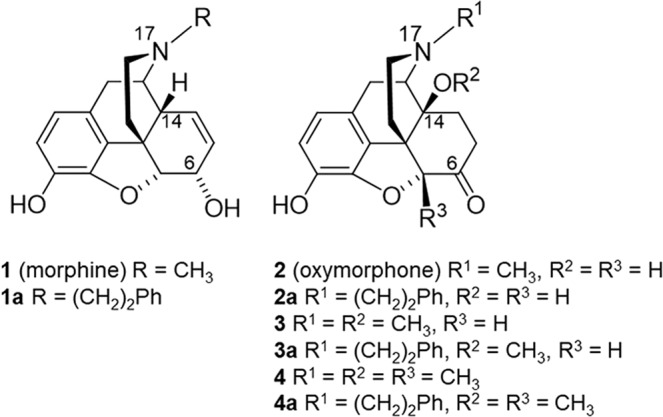
Structures of *N*-methylmorphinans **1–4** and their *N*-phenethyl counterparts **1a**–**4a**. Ph, phenyl.

Morphine (**1**, Fig. 1), the prototypical opioid, has been used for decades for pain relief, and its addictive properties are long and well recognized. Over the years, numerous semisynthetic and synthetic investigations were reported aiming at optimizing morphine’s biological actions, especially its safety profile^1–3^. These studies have resulted in clinically useful drugs for the treatment of pain and other human disorders (drug abuse, alcohol abuse, and gastrointestinal motility dysfunction), as well as in research tools^1–5^. Morphine and structurally-derived compounds (e.g. oxycodone, oxymorphone, hydromorphone) are agonists at the µ opioid receptor (µOR), a G protein-coupled receptor (GPCR), and the opioid receptor subtype that primarily mediates desirable (analgesia) but also undesirable effects (i.e. constipation, respiratory depression, sedation, analgesic tolerance and dependence) of opioids^4–6^. Moreover, the number of people misusing opioids, as well as of opioid-related deaths have increased dramatically during the past years^7^.

The *N*-phenethyl substituted derivative of morphine (**1a**, Fig. 1) was prepared by Clark *et al*. and Small *et al*. in the 1950s^8,9^, and the *N*-phenethyl substituted derivative of oxymorphone (**2a**, Fig. 1) was prepared by Seki *et al*. in the 1960s^10^, with both morphinan opioids having increased antinociceptive potency in mice than their *N*-methyl analogues^11,12^. We have reported the *in vitro* profile of **1a** and **2a**, including binding and activation of the µOR, and were first to show that the presence of an *N*-phenethyl group at position 17 is highly favorable in terms of improved affinity and selectivity at the µOR and potent µOR agonism *in vitro*^13^.

The hydroxyl group at position 14 is known to play a critical role in the agonist activity *in vitro* and *in vivo* of *N*-methylmorphinan-6-ones^2,3,14,15^. The 14-methoxy substituted analogues of oxymorphone (**2**), namely 14-*O*-methyloxymorphone (14-OMO, **3**, Fig. 1)^16^ and 14-methoxymetopon (14-MM, **4**, Fig. 1)^17^ show increased µOR affinity and agonism, efficacy and potency than oxymorphone (**2**)^16–18^. Whereas **3** induces the usual morphine-like adverse effects, **4** has a superior benefit/risk ratio^15,18^. In this study, we describe the synthesis and pharmacological evaluation of *N*-phenethyl substituted derivatives **3a** and **4a** (Fig. 1). We have also aimed to investigate the effect of the replacement of the *N*-methyl group in 14-OMO (**3**) and 14-MM (**4**) by an *N*-phenethyl substituent in **3a** and **4a**, respectively, on *in vitro* profiles (opioid receptor binding and functional activities), and *in vivo* behavioural properties (nociception and motor function) in mice. Furthermore, the current work was undertaken to understand the consequences of the substitution of the *N-*methyl group in *N*-methylmorphinans **1**–**4** by an *N*-phenethyl group in **1a**–**4a** on their pharmacological activities using molecular docking and molecular dynamics (MD) simulations, to gain insights on their binding and subtype profile for opioid receptors. The emerged structure-activity relationships (SARs) in this class of opioid morphinans provide essential information for tuning functional *in vitro* and *in vivo* activities towards discovery of effective and safer analgesics for the pain treatment.

## Results and Discussion

### Chemistry

**Scheme 1:**
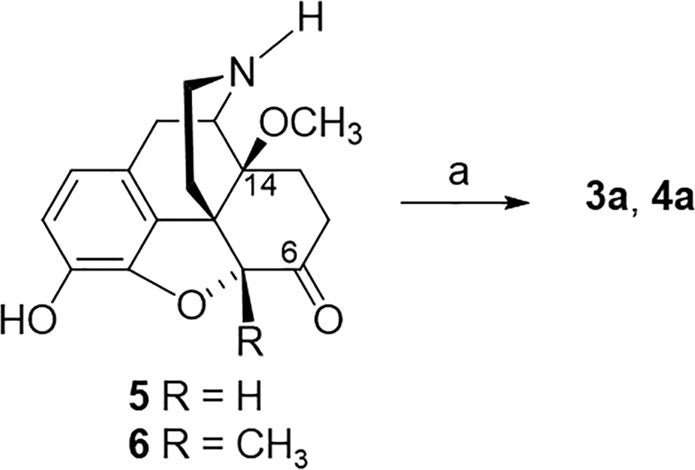
Synthesis of Compounds **3a***ª* and **4a**^*a a*^Reagents and conditions: (a) phenethyl bromide, NaHCO_3_, DMF, 80 °C, 48 h.

The new *N*-phenethylmorphinans **3a** and **4a** were prepared from their precursors **5**^19^ and **6**, respectively, by N-alkylation with phenethyl bromide as presented in Scheme 1. The synthesis of *N*-phenethylmorphinans **1a** and **2a** has been earlier reported^8–10^, with some modifications as described^13^.

### Pharmacological evaluation

Binding affinities at the human µOR, δ (δOR) and κ (κOR) opioid receptors were first determined in *in vitro* competition binding assays using membranes from Chinese hamster ovary (CHO) cells stably transfected with one of the recombinant human opioid receptors (CHO-hµOR, CHO-hδOR and CHO-hκOR cells) as previously described^13,20^. We have reported earlier that *N*-phenethyl substituted morphinans **1a** and **2a** have higher affinities at the µOR in the rat brain than their parent *N*-methylmorphinans morphine (**1**) and oxymorphone **2**^13^. Herein, we have evaluated their binding profile at the human µOR expressed in CHO cells, and made similar observations with **1a** and **2a** displaying ca. 13- and 12-fold increased affinity at the human µOR than **1** and **2**, respectively (Table 1). In this study, comparison of the *in vitro* binding at the µOR of 14-methoxy-*N*-methylmorphinan-6-ones to their *N*-phenethyl analogues revealed that introduction of a phenethyl group at position 17 does not influence affinity at the µOR when relating 14-OMO (**3**) vs. **3a**, and 14-MM (**4**) vs. **4a** (*P* > 0.05, t-test). Furthermore, all *N*-phenethyl derivatives **1a–4a** have higher affinities (5- to 13-fold) than their *N*-methyl counterparts **1**–**4** at the human δOR (*P* < 0.05, t-test). Particularly, **3a** and **4a** showed very low nanomolar affinities at the δOR (K_i_ = 1.81 nM and 1.45 nM, respectively). While affinities of **1a**, **2a** and **3a** at the human κOR were in the range of their parent molecules **1–3**, a ca. 3-fold decrease in the κOR affinity was noted for **4a** vs. **4** (Table 1). We have also observed that replacement of the *N*-methyl group in **1** and **2** with an *N*-phenethyl group enhanced not only µOR affinity but also µOR selectivity vs. δOR and vs. κOR of **1a** and **2a**. In the case of 14-methoxy-*N*-phenethylmorphinan-6-ones **3a** and **4a**, a reduction in µOR vs. δOR selectivity was noticed, while selectivity for µOR vs. κOR was higher than that of 14-OMO (**3**) and 14-MM (**4**), respectively (Table 1).

**Table 1:** Comparison of Binding Affinities at Opioid Receptors of *N*-Methylmorphinans **1–4** and Their *N*-Phenethyl Analogues **1a**–**4a**.

Compound	K_i_ (nM)^a^				
	µOR	δOR	κOR	K_i_ ratio δOR/µOR	K_i_ ratio κOR/µOR
**Morphine (1)**	3.35 ± 0.30	195 ± 26	96.4 ± 0.5	58	29
**1a**	0.25 ± 0.09***	24.5 ± 8.7*	93.5 ± 2.9	98	374
**Oxymorphone (2)**	1.41 ± 0.30	79.1 ± 9.3	32.6 ± 9.8	56	23
**2a**	0.12 ± 0.07*	10.7 ± 5.1*	42.2 ± 7.3	89	352
**14-OMO (3)**	0.27 ± 0.09	9.08 ± 0.31	10.3 ± 1.8	34	38
**3a**	0.19 ± 0.02	1.81 ± 0.68***	15.8 ± 8.8	9.5	83
**14-MM (4)**	0.25 ± 0.04	18.6 ± 0.98	12.8 ± 1.5	74	51
**4a**	0.24 ± 0.03	1.45 ± 0.31***	35.3 ± 5.5**	6.0	147

^a^Determined in competition binding assays using membranes from CHO cells stably expressing the human opioid receptors. Values represent the mean ± SEM (n = 3–4). *P < 0.05, **P < 0.01 and ***P < 0.001 for *N*-methylmorphinans vs. respective *N*-phenethyl analogues (unpaired t-test).

*In vitro* opioid activities of targeted compounds at the human µOR and δOR were determined in the guanosine-5′-*O*-(3-[^35^S]thio)-triphosphate ([^35^S]GTPγS) binding (Table 2) and forskolin-induced cAMP accumulation assays (Table 3), performed as described^13,21^. The κOR-mediated G protein activation was assessed using [^35^S]GTPγS binding assays with CHO cell membranes expressing the human κOR (Table 2). Previous work from our laboratory on the introduction of a phenethyl group at the nitrogen in morphine (**1**) and oxymorphone (**2**) showed an increase in agonist potency by 2- to 3-fold and full efficacy for **1a** and **2a** in inducing µOR-mediated G protein signaling as assessed by [^35^S]GTPγS binding and calcium mobilization assays^13^. Similarly, enhanced µOR agonist potencies by 4- to 5-fold were measured for *N*-phenethyl analogues **1a** and **2a** as compared to morphine and oxymorphone, respectively, in the cAMP accumulation assay, while the δOR agonism remained unchanged (Table 3). In the series of 14-*O*-methylmorphinan-6-ones, exchanging the *N*-methyl by an *N*-phenethyl substituent did not largely influence agonist potency and full efficacy at the µOR of 14-OMO (**3**) vs. **3a**, and 14-MM (**4**) vs. **4a** (*P* > 0.05, t-test). These findings reveal that the *N*-phenethyl substitution in 14-methoxy-*N*-methylmorphinan-6-ones does not cause any change in binding affinity nor *in vitro* agonism at the µOR. All compounds displayed full efficacies at the δOR with different levels of potencies, while at the κOR a partial agonist profile with very low potencies was noted (Tables 2 and 3). In the [^35^S]GTPγS binding assay, the 14-methoxy-*N*-phenethylmorphinan-6-ones **3a** and **4a** displayed the highest agonist potencies at the δOR (EC_50_ = 9.34 nM and 9.54 nM, respectively), which were higher (4-fold) than potencies of their *N*-methyl counterparts 14-OMO (**3**) and 14-MM (**4**). The same observation was made when comparing agonist activity of **3** vs. **3a** and **4** vs. **4a** at the δOR in the cAMP accumulation assay, with a significant increase in potency (*P* < 0.05, t-test) for the *N*-phenethylmorphinan-6-ones **3a** and **4a** (Table 3). Thus, the outcomes from the [^35^S]GTPγS functional and cAMP accumulation assays are in agreement with the results on increased binding affinity at the δOR of **3a** and **4a** compared to **3** and **4**, respectively. Additionally, **3a** and **4a** have a functional profile *in vitro* as dual µ/δOR full agonists, a class of ligands nowadays targeted as new analgesics with reduced unwanted side effects. Numerous pharmacological and biochemical reports and studies with opioid receptor knockout mice have provided evidence on the modulatory interactions between the µOR and δOR systems^22–26^. While the mechanisms are still unknown, several studies have established that the therapeutic profile of opioids could be improved by simultaneous modulation of the µOR and δOR, with compounds designed to target both receptors based on peptidic structures, non-peptidic structures or utilize the morphinan scaffold^25–32^.

**Table 2:** Comparison of Functional Activities at Opioid Receptors of *N*-Methylmorphinans **1–4** and Their *N*-Phenethyl Analogues **1a**–**4a**.

Compound	µORa		δORa		κORa	
	EC_50_ (nM)	% stim.	EC_50_ (nM)	% stim.	EC_50_ (nM)	% stim.
**Morphine (1)**	34.4 ± 5.1^*b*^	89 ± 17^*b*^	668 ± 65^*b*^	109 ± 14^*b*^	710 ± 23^*b*^	76 ± 2^*b*^
**1a**	10.3 ± 0.9^b^*	113 ± 8^*b*^	712 ± 86^*b*^	138 ± 17^*b*^	1049 ± 29^*b*^	19 ± 2^b^***
**Oxymorphone (2)**	7.80 ± 1.61^*b*^	92 ± 5^*b*^	259 ± 33^*b*^	87 ± 40^*b*^	463 ± 116^*b*^	48 ± 11^*b*^
**2a**	2.67 ± 1.06^b^*	97 ± 3^*b*^	131 ± 60^*b*^	101 ± 9^*b*^	225 ± 74^*b*^	7.5 ± 0.01^b^*
**14-OMO (3)**	1.21 ± 0.48	95 ± 5	38.5 ± 6.9	102 ± 4	135 ± 29	65.9 ± 6.5
**3a**	1.26 ± 0.63	98 ± 10	9.34 ± 0.60*	107 ± 5	144 ± 9	35.4 ± 7.5*
**14-MM (4)**	2.66 ± 0.58	99 ± 5	36.8 ± 12.4	100 ± 9	181 ± 9	68.9 ± 9.2
**4a**	1.86 ± 0.84	102 ± 13	9.54 ± 2.33*	103 ± 2	334 ± 114	51.3 ± 10.4

^a^Determined in [^35^S]GTPγS binding assays using membranes from CHO cells stably expressing the human opioid receptors. Percentage stimulation (% stim.) relative to the agonist DAMGO (µOR), DPDPE (δOR) or U69,593 (κOR). Values represent the mean ± SEM (n = 3–4). ^*b*^Data from ref. ^13^. *P < 0.05, **P < 0.01 and ***P < 0.001 for *N*-methylmorphinans vs. respective *N*-phenethyl analogues (unpaired t-test).

**Table 3:** Agonist potencies and efficacies at the human µOR and δOR of *N*-methylmorphinans **1**–**4** and their respective *N*-phenethy analogues **1a**–**4a** in the cAMP accumulation assay.

Compound	µOR^a^		δOR^a^	
	EC_50_ (nM)	% stim.	EC_50_ (nM)	% stim.
**Morphine (1)**	13.5 ± 2.83	106 ± 9	374 ± 24	94 ± 18
**1a**	2.87 ± 0.91*	107 ± 5	315 ± 110	105 ± 8
**Oxymorphone (2)**	2.48 ± 0.79	109 ± 2	56.9 ± 5.1	87 ± 16
**2a**	0.59 ± 0.04*	97 ± 6	50.1 ± 16.2	99 ± 18
**14-OMO (3)**	0.19 ± 0.14	90 ± 8	5.42 ± 0.93	98 ± 3
**3a**	0.078 ± 0.004	88 ± 12	0.60 ± 0.07**	93 ± 2
**14-MM (4)**	0.31 ± 0.07	93 ± 5	4.06 ± 0.89	99 ± 8
**4a**	0.15 ± 0.07	98 ± 12	0.55 ± 0.16*	96 ± 4

^a^Determined in the forskolin-induced cAMP accumulation assay using CHO cells co-expressing the human opioid receptors and the cAMP biosensor GloSensor-22F (CHO-hµOR-p22F or CHO-hδOR-p22F cells). Percentage stimulation (% stim.) relative to the agonist DAMGO (µOR) or DPDPE (δOR). Values represent the mean ± SEM (n = 3–4). *P < 0.05 and **P < 0.01 for *N*-methylmorphinans vs. respective *N*-phenethyl analogues (unpaired t-test).

**Figure 2:**
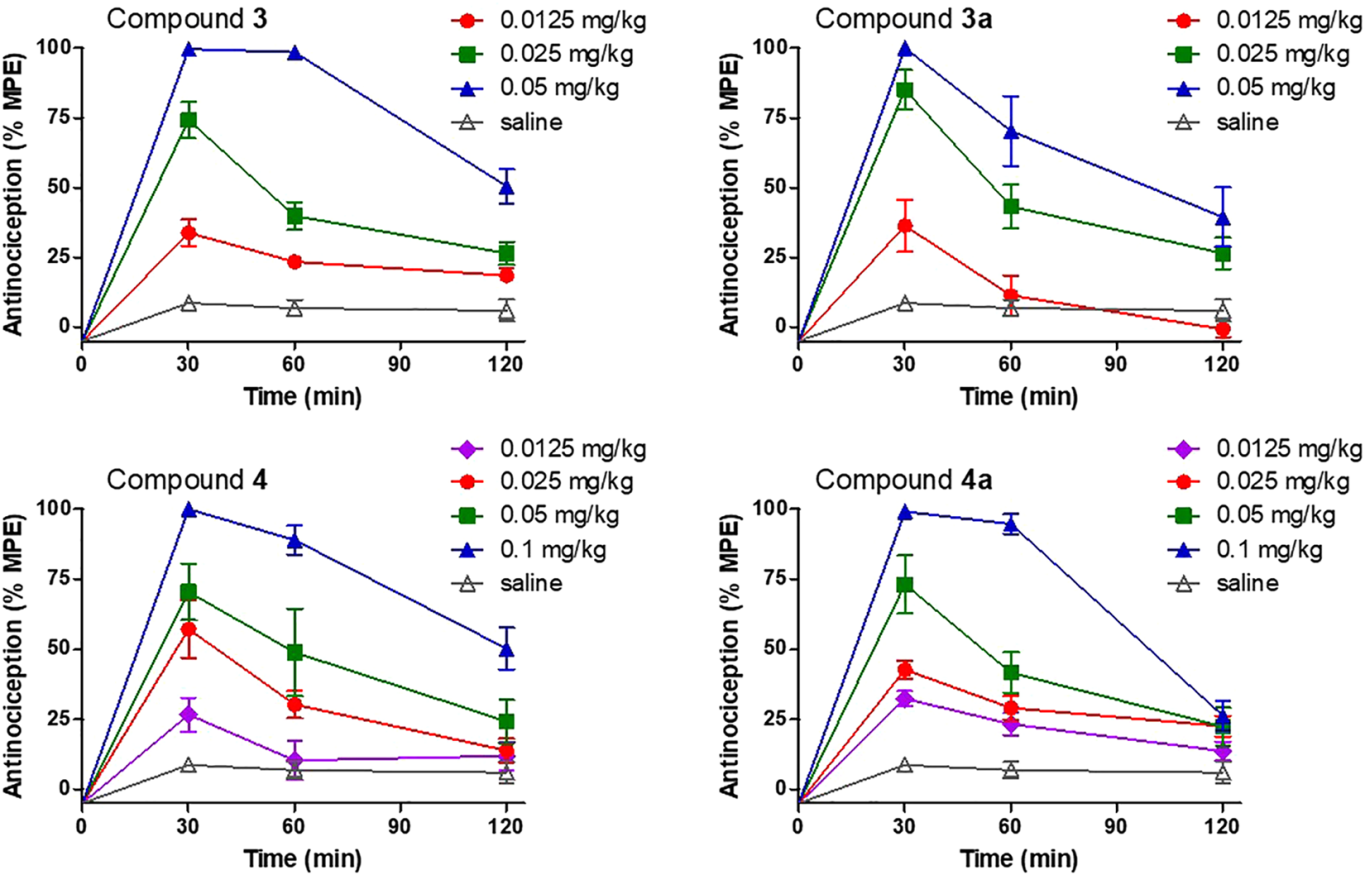
Time- and dose-dependent antinociceptive effects of 14-methoxy-*N*-methylmorphinan-6-ones 14-OMO (**3**) and 14-MM (**4**) and their respective *N*-phenethyl analogues **3a** and **4a** in the tail-flick assay in mice after s.c. administration. Data are shown as the mean %Maximum Possible Effect (%MPE) ± SEM (n = 5–6 mice per group).

**Figure 3:**
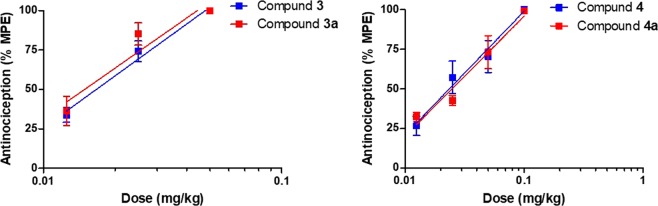
Comparison of dose-dependent antinociceptive effects of 14-methoxy-*N*-methylmorphinan-6-ones 14-OMO (**3**) and 14-MM (**4**) and their respective *N*-phenethyl analogues **3a** and **4a** in the tail-flick assay in mice at 30 min after s.c. administration. Data are shown as mean %Maximum Possible Effect (%MPE) ± SEM (n = 5–6 mice per group). *P* > 0.05 for **3** vs. **3a**, and **4** vs. **4a** (two-way ANOVA).

**Figure 4:**
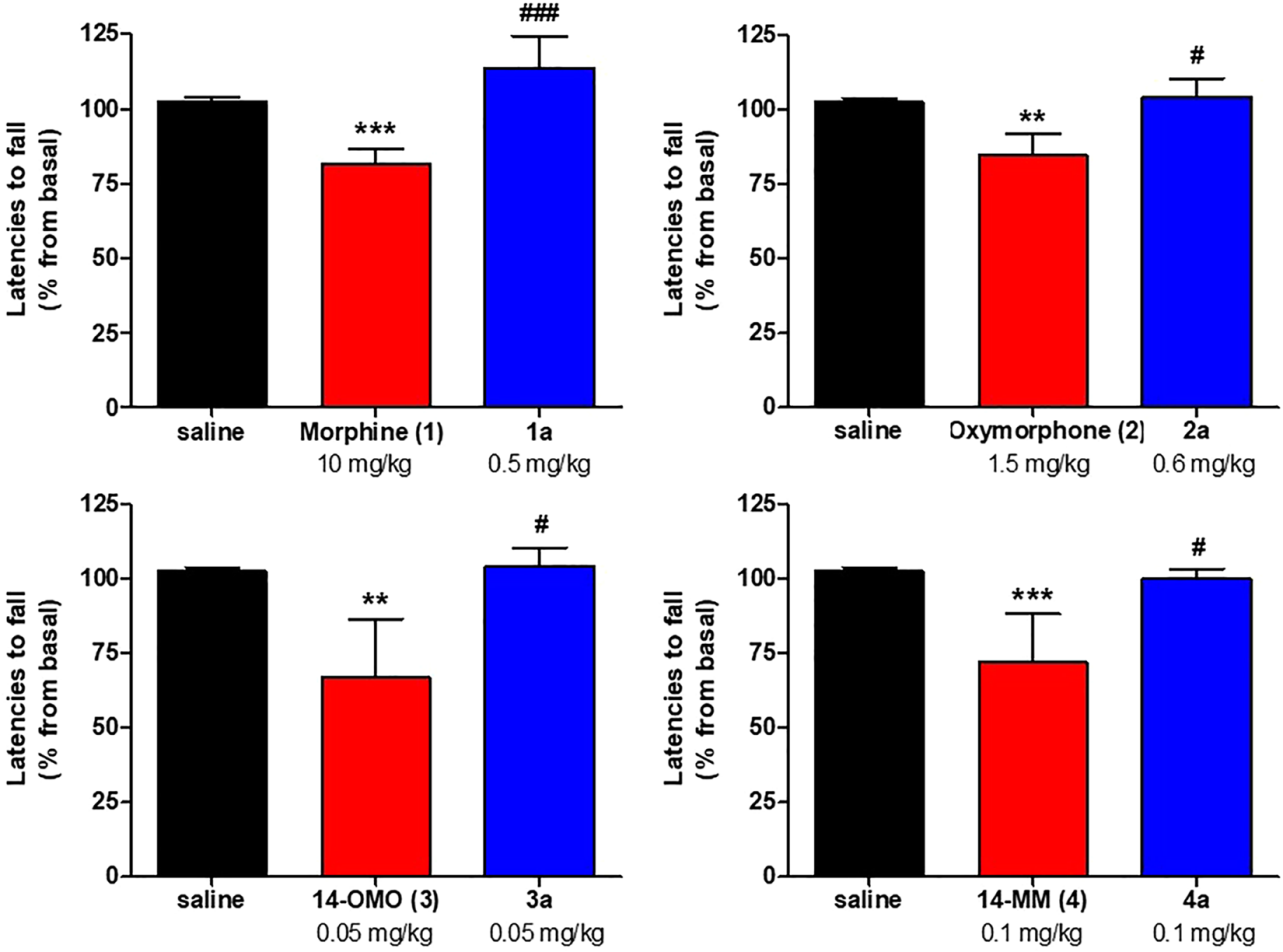
Effect of *N*-methylmorphinans **1**–**4** and their respective *N*-phenethyl analogues **1a**–**4a** in the mouse rotarod assay. Mice were tested 30 min after s.c. administration of control (saline) or test compounds. Data depicts latencies to fall from the rotarod as the mean percent changes from baseline performance ± SEM (n = 6 mice per group). *P < 0.05, **P < 0.01 and ***P < 0.001 vs. saline group; ^#^*P* < 0.05 and ^###^*P* < 0.001 vs. *N*-methylmorphinan treated group; one-way ANOVA followed by Tukey’s *post hoc* test.

We^13^ and others^11,12^ have reported that the *N*-phenethyl substituted morphinans **1a** and **2a** exhibit increased antinociceptive potencies than their respective *N*-methyl analogues morphine (**1**) and oxymorphone (**2**) in mouse models of acute thermal nociception after subcutaneous (s.c.) administration, which is in line with findings from binding and functional *in vitro* assays. We have shown that **1a** was 22- and 28-fold more effective than morphine (**1**) in the hot-plate and tail-flick tests, respectively^13^. Further, the *N*-phenethyl analogue of oxymorphone (**2a**) was found to be highly active with about 2-fold higher potency than oxymorphone (**1**) (Table 4)^13^. In this study, we have also evaluated if targeted structural changes in 14-OMO (**3**) and 14-MM (**4**) also affects antinociceptive activities. Antinociceptive effects of *N*-phenethyl substituted **3a** and **4a** were assessed in the tail-flick assay in mice after s.c. administration as described^13^. Antinociceptive potencies (ED_50_ values) were determined at the peak of action and compared to those of *N*-methyl counterparts **3** and **4** (Table 4). All compounds increased tail-withdrawal latencies to thermal stimulation in a time- and dose-dependent manner with a peak effect generally occurring at 30 min (Fig. 2). As shown in Table 4 and Fig. 3, **3a** and **4a** display similar antinociceptive activities to their analogues 14-OMO (**3**) and 14-MM (**4**), respectively (*P* > 0.05, two-way ANOVA), indicating that exchanging the *N*-methyl by an *N*-phenethyl group in 14-*O*-methylmorphinan-6-ones does not affect the *in vivo* agonism.

**Table 4:** Antinociceptive Potencies of *N*-Methylmorphinans **1–4** and Their *N*-Phenethyl Analogues **1a**–**4a** in the Tail-Flick Test in Mice.

Compound	ED_50_ (mg/kg, s.c.) (95% CL)^*a*^
**Morphine (1)**	3.06 (1.76–5.31)^*b*^
**1a**	0.11 (0.027–0.40)^*b*^
**Oxymorphone (2)**	0.35 (0.16–0.77)^*b*^
**2a**	0.15 (0.058–0.40)^*b*^
**14-OMO (3)**	0.014 (0.0051–0.037)
**3a**	0.014 (0.0086–0.023)
**14-MM (4)**	0.024 (0.0093–0.062)
**4a**	0.024 (0.0091–0.062)

^*a*^ED_50_ values and 95% confidence intervals (CL in parentheses) were calculated at 30 min (peak effect) from dose-response curves (n = 5–6 mice per group). ^*b*^Data from ref. ^13^.

Clinically used opioid analgesics, such as morphine, oxycodone or fentanyl, are known to produce sedation and motor dysfunction, side effects that limits their clinical usefulness^33–35^. With literature evidence that mixed µOR/δOR agonists are efficacious analgesics with reduced side effects^25–32^, we have evaluated the effect of **3a** and **4a** as mixed µ/δOR agonists, and behavioral consequences of the replacement of the *N-*methyl group in *N*-methylmorphinans **1**–**4** by an *N*-phenethyl group in **1a–4a** on motor coordination in mice using the rotarod assay, a well-established model for evaluating loss of coordinated locomotion^36^. The first behavioral data on motor function following systemic s.c. administration of *N*-phenethyl substituted derivatives of morphine and oxymorphone, **1a** and **2a**, respectively are presented. Mice were s.c. treated with the respective compound at doses equivalent to a 3- to 4-fold the antinociceptive ED_50_ dose. Rotarod experiments demonstrate the lack of the mixed µ/δOR agonists **3a** and **4a** to induce motor dysfunction, having an improved profile than their parent µOR selective agonists 14-OMO (**3**) and 14-MM (**4**), respectively (Fig. 4). Whereas morphine (**1**) and oxymorphone (**2**) caused a significant deficit in rotarod performance, their *N*-phenethyl substituted **1a** and **2a** did not affect the evoked locomotor activity of mice (Fig. 4). In this study, we show that *N*-phenethyl substituted morphinans **1a–4a** elicit effective and potent antinociception without motor impairment in mice.

### Molecular modeling

**Figure 5:**
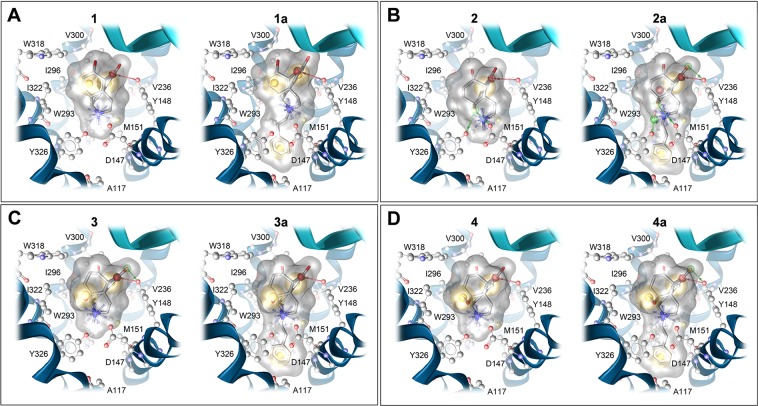
Predicted binding modes at the µOR (PDB ID: 5C1M) and receptor-ligand interaction patterns of *N*-methylmorphinans **1**–**4** and their respective *N*-phenethyl analogues **1a**–**4a**. (**A**) Morphine (**1**) and **1a**, (**B**) oxymorphone (2**)** and **2a**, (C) 14-OMO (**3**) and **3a**, and (D) 14-MM (**4**) and **4a**. Yellow spheres indicate lipophilic contacts, red arrows hydrogen bond acceptors, green arrows hydrogen bond donors and positively charged centers are shown as blue spheres.

The µOR was the first opioid receptor type resolved in an inactive (PDB ID: 4DKL)^37^ and an active conformation (PDB ID: 5C1M)^38^. The access to crystal structures of the µOR provides essential knowledge on key aspects of the µOR pharmacology and its function^37–39^. All investigated morphinans (Fig. 1) bind and are agonists at the µOR. The observed similarities or differences in their *in vitro* and *in vivo* activity profiles incited exploration of their binding modes at the µOR. Molecular docking investigations were performed with *N*-methylmorphinans **1–4** and their *N*-phenethyl counterparts **1a–4a**, where a 3D-pharmacophore approach based on the LigandScout program^29^ was applied to analyze shared and distinct receptor-ligand interactions. Docking studies using the active conformation of the µOR (PDB ID: 5C1M)^38^ revealed comparable binding orientations for all targeted morphinans, which are in accordance with BU72^27^ in its co-crystallized conformation. An overview of detected interactions is presented in Figs. 5 and S1.

Although all investigated compounds show a comparable binding mode to the µOR in which the morphinan moiety was found to adopt a similar orientation and to share several essential receptor-ligand interactions, we have also observed interaction pattern differences related to specific structural features (Fig. 5). The tertiary amine forms an essential charge interaction with D147 and a π–cation interaction with Y148 residue. The crucial role of D147 for the binding to the µOR of morphinan ligands, as well as other chemotypes (i.e. mitragynine pseudoindoxyl) and peptides (i.e. DAMGO) has been described^37,38,41–44^. The interaction with Y148 is also recognized as an important requirement for ligands (small molecules and peptides) to bind to the µOR^37,38,41–44^. In this study, the oxygen of the partially saturated furan ring (E-ring) of the morphinan system serves as a hydrogen bond acceptor for Y148 in both series of *N*-methyl (**1**–**4**) and *N*-phenethyl substituted morphinans (**1a–4a**). The 14-*O*-methylmorphinan-6-ones **3**, **4**, **3a** and **4a** show a lipophilic contact of the 14-methoxy group with I322 (Fig. 5C,D). The phenolic substructure lies opposite to M151, V236 and V300 residues. The 14-hydroxyl group of oxymorphone (**2**) and its *N*-phenethyl analogue **2a** forms hydrogen bonds to both D147 and Y326 residues (Fig. 5B). Compounds **1** and **1a** exhibited the same interaction pattern with the only difference in the additional lipophilic contacts of the *N*-phenethyl moiety of **1a** with a lipophilic subpocket (Fig. 5A). For all ligands with an *N*-phenethyl group, this moiety is embedded in a lipophilic pocket formed by A117, W293 and Y326 residues.

**Figure 6:**
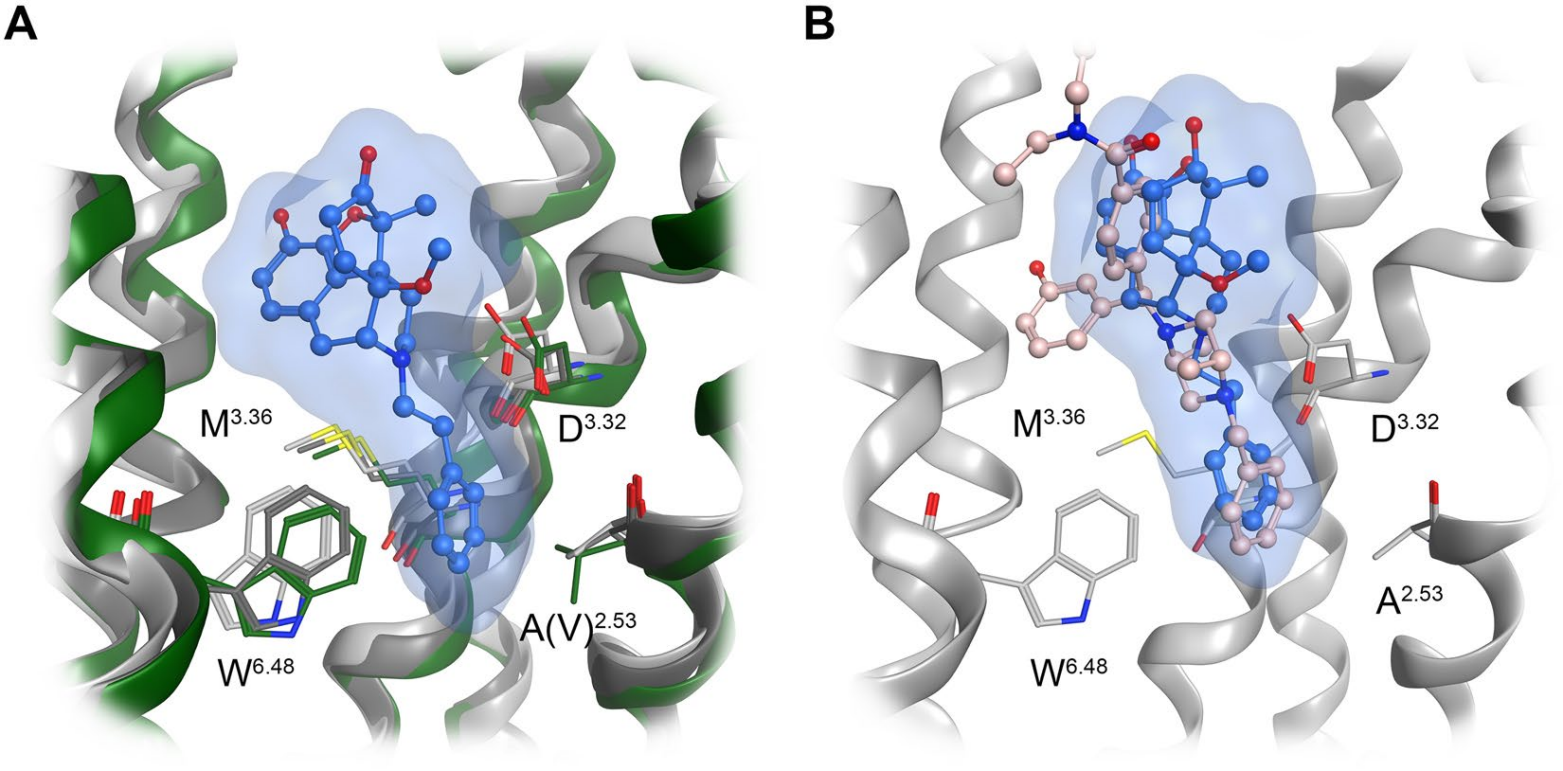
Structural comparison of the orthosteric binding pocket of the µOR (PDB ID: 5C1M; dark grey), δOR (PDB ID: 6PT3; light grey) and κOR (PDB ID: 6B73, green), with **4a** in its µOR bound conformation (**A**). Size and shape of the lipophilic subpocket formed by A^2.53^, M^3.36^ and W^6.48^, which hosts the *N*-phenethyl substituent of **4a** is different at the κOR (green), due to a valine instead of an alanine residue. (**B**) The proposed binding mode of **4a** (blue) at the δOR (PDB ID: 6PT3) shows a similar orientation of the *N*-phenethyl substituent compared to the phenyl ring of the co-crystallized agonist DPI-287 (salmon).

The recent crystal structure of the δOR (PDB ID: 6PT3)^45^ with the co-crystallized agonist DPI-287 supports our proposed binding mode of **4a** at the δOR, since DPI-287 also has a phenyl ring which is filling the beforementioned lipophilic subpocket (Fig. 6). The active κOR structure (PDB ID: 6B73) was also determined in complex with the epoxymorphinan agonist MP1104^46^. Compared to the µOR, the size and shape of this subpocket was found to be highly similar for the δOR (Figs. 6A and S2), but different for the κOR (Figs. 6 and S2). Whereas µOR and δOR have an alanine residue in position 2.53 (according to Ballesteros-Weinstein nomenclature), a valine residue at the same position requires more space in the κOR resulting in a smaller lipophilic subpocket (Fig. S2). We suggest that this structural difference is important for the subtype selectivity of targeted *N*-phenethyl substituted morphinans and the resulting dual µ/δOR activity of **3a** and **4a**.

**Figure 7:**
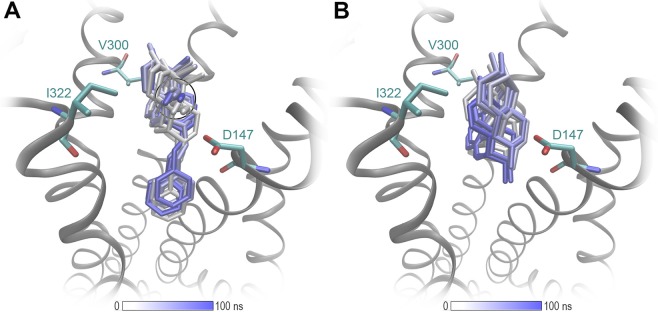
MD simulations of the µOR (PDB ID: 5C1M) support the docking results. (**A**) The binding mode of **4a** is stabilized by a lipophilic contact I322 with the 14-*O*-methyl group (black circle) and furthermore the *N*-phenethyl moiety. (**B**) **1** shows more fluctuations in its binding mode, due to the absence of these interactions.

Several studies have evidenced MD simulations as an effective approach to examine binding modes between opioid receptors and their ligands^39,41–44^. In order to further validate the binding modes of morphinans **1**–**4** and **1a–4a** depicted using molecular docking, we performed all-atoms MD simulations of the µOR as described^47^. The analysis of MD simulations support the docking results and show that the binding location, the major ligand orientation in the binding pocket and the key interactions reported from the docking experiments remain firm over 100 ns of MD simulations (Figs. 7 and S3).

All investigated ligands are full agonists at the µOR, in accordance with the observation that all structures provide a full constriction of the orthosteric binding site as a key feature for receptor activation. The additional lipophilic contacts of the *N*-phenethyl substituent deep in the core region of the receptor are supposed to enhance ligand binding. This effect is less prominent for morphinans in which the parent compound already has a binding affinity in the subnanomolar range to the µOR (Table 1). This may be explained by additional interactions with the receptor, such as additional hydrogen bonds for the 14-hydroxymorphinans **2** and **2a**, or an additional lipophilic contact with I322 residue for the 14-*O*-methylmorphinans, 14-OMO (**3**), 14-MM (**4**), **3a** and **4a**. The latter interaction is of particular importance for orientation of the ligand in the binding site, and is visualized in Fig. 7. Interestingly, *N*-methyl substituted **1** and **2** show a slightly different orientation compared with their related *N*-phenethyl analogues (Fig. S4). This effect is not observable for **3**, **3a**, **4** and **4a**, which suggests that an increased µOR affinity of morphinan analogs can be achieved by either a methoxy substitution at position 14 or by an *N*-phenethyl group. Since the combination of the two strategies does not show additive effects, we assume that the optimal orientation can be sufficiently triggered by only one of the two substitutions. Furthermore, a direct comparison of the active δOR^45^ and the µOR^38^ crystal structures unveils the high similarity of their binding pockets. While all residues forming key interactions are identical, a major difference was observed at the beginning of helix seven at position 7.35 (Fig. S5). The tryptophan residue in µOR was found to be optimal in forming lipophilic contacts with the morphinan moiety, whereas this receptor-ligand contact is missing for the δOR, due to a leucine at this position. This might explain why all studied compounds are have higher affinity for the µOR compared to the δOR.

## Conclusions

The results of the present study provide SAR evidence on the consequences of an *N-*methyl substitution in morphinan opioids **1–4** by an *N*-phenethyl in **1a–4a** on *in vitro* and *in vivo* activities, with molecular docking and MD simulations studies offering a structural basis for the observed pharmacological profiles at the opioid receptors. Pharmacological findings are supported by docking and MD simulations analysis with *N*-methyl substitute morphine (**1**) and oxymorphone (**2**) showing a slightly different orientation in the binding pocket of the µOR compared to their related *N*-phenethyl analogues, **1a** and **2a**, respectively. This was not noticed for 14-OMO (**3**) vs. **3a**, and 14-MM (**4**) vs. **4a**, indicating that an increased µOR affinity can be achieved by either a 14-methoxy or by an *N*-phenethyl substitution, as key sites to be targeted in modulating the binding affinity and efficacy of morphinans to the µOR. Whereas replacement of the *N*-methyl substituent in morphine (**1**) and oxymorphone (**2**) by an *N*-phenethyl group enhanced binding affinity, selectivity and agonist potency at the µOR of **1a** and **2a**, the *N-*phenethyl substitution in 14-methoxy-*N*-methylmorphinan-6-ones (**3** and **4**) turned selective µOR ligands into dual µ/δOR agonists (**3a** and **4a**), a profile that currently emerges as a promising approach to opioid analgesic drug discovery^26–32^. Furthermore, we have demonstrated that the *N*-phenethyl substituted morphinans **1a–4a** are effective and potent antinociception agents without causing unwanted motor impairment in mice after s.c. administration. Altogether, these data offer important insights on the SARs in the morphinan class of opioid ligands, by increasing the current understanding of the impact of different substituents at the nitrogen and position 14 on ligand-µOR binding, receptor activation and the link between antinociception and side effects (i.e. motor function).

## Materials and Methods

### Chemistry

General chemical and analytical methods were performed according to protocols as described previously^20^. All chemicals were of reagent grade and obtained from standard commercial sources. Melting points were determined on a Kofler melting point microscope and are uncorrected. ^1^H and ^13^C NMR spectra were recorded on a Bruker Avance II + spectrometer operating at 600 MHz and equipped with a Prodigy TCI probe. IR spectra were taken on a Bruker Alpha FT-IR spectrometer (for detection, an ATR sensor was used). Mass spectra were recorded on a Varian MAT 44 S apparatus. Elemental analyses were performed at the Microanalytic Laboratory of the University of Vienna, Austria. For column chromatography (MPLC), silica gel 60 (0.040–0.063 mm, Fluka, Switzerland) was used. Compounds **3a** and **4a** were used as bases for testing. The combustion analysis values were found to be within ± 0.4% of the calculated values, confirming a purity of the tested compounds of >95%.

### Synthesis of 4,5α-Epoxy-3-hydroxy-14-methoxy-N-phenethylmorphinan-6-one (3a)

A mixture 4,5α-epoxy-3-hydroxy-14-methoxymorphinan-6-one hydrochloride (**5** ∙ **HCl**) (100 mg, 0.296 mmol), prepared according to the described procedure^19^, phenethyl bromide (76.7 mg, 0.40 mmol), and NaHCO_3_ (67.3 mg, 0.8 mmol) in 3 mL DMF was stirred at 80 °C for 48 h. The cooled mixture was filtered, the filtrate evaporated to dryness and purified by column chromatography (CH_2_Cl_2_/MeOH/NH_4_OH 97.5/1.5/1) to yield 40 mg (47%) of compound **3a**. Mp 180–182 °C. IR (ATR) 2929 (OH), 1718 (CO) cm^−1^; ^1^H NMR (CDCl_3_): δ 7.25–7.14 (m, 5 arom. H), 6.73 (d, *J* = 8.0 Hz, *H*-C(1)), 6.62 (d, *J* = 8.0 Hz, *H*-C(2)), 4.68 (s, *H*-C(5)), 3.13 (s, *CH*_3_O); ^13^C NMR (CDCl_3_): δ 209.54, 143.40, 140.37, 138.68, 129.34, 128.83, 128.37, 126.10, 125.08, 119.85, 117.57, 90.56, 75.61, 56.98, 56.33, 51.03, 47.96, 44.23, 35.50, 34.42, 29.17, 24.97, 23.44; MS (ESI) *m/z* 406.3 [M + 1]^+^. Anal. (C_25_H_27_NO_4_ ∙ 0.2CH_2_Cl_2_ ∙ 0.1MeOH) C, H, N.

### Synthesis of 4,5α-Epoxy-3-hydroxy-14-methoxy-5-methyl-N-phenethylmorphinan-6-one (4a)

A mixture 4,5α-epoxy-3-hydroxy-14-methoxy-5-methylmorphinan-6-one hydrobromide (**6** ∙ **HBr**) (100 mg, 0.23 mmol), phenethyl bromide (74.3 mg, 0.41 mmol), NaHCO_3_ (66.8 mg, 0.8 mmol), in 2.5 mL DMF was stirred at 80 °C for 48 h. The mixture was cooled and filtered, the filtrate evaporated to dryness and the crude product purified by column chromatography (CH_2_Cl_2_/MeOH/NH_4_OH 97/2/1) to yield 46 mg (38%) of compound **4a**. Mp 179–180 °C. IR (ATR) 2920 (OH), 1718 (CO) cm^−1^. ^1^H NMR (CDCl_3_): δ 7.31–7.25 (m, 5 arom. H), 6.70 (d, *J* = 8.1 Hz, *H*-C(1)), 6.57 (d, *J* = 8.1 Hz, *H*-C(2)), 3.17 (s, *CH*_3_O), 1.55 (s, *CH*_3_-C(5)); ^13^C NMR (CDCl_3_): δ 213.01, 143.11, 140.37, 138.36, 128.80, 128.39, 126.10, 119.47, 117.35, 96.48, 76.20, 56.98, 56.38, 51.39, 47.50, 43.91, 34.56, 34.38, 25.86, 25.15, 23.44, 17.43; MS (ESI) *m/z* 420.3 [M + 1]^+^. Anal. (C_26_H_29_NO_4_ ∙ 0.5CH_2_Cl_2_) C, H, N.

### Synthesis of 4,5α-Epoxy-3-hydroxy-14β-methoxy-5-methylmorphinan-6-one hydrobromide (6 ∙ HBr)

A solution of 3,14-dimethoxy-4,5α-epoxy-5β-methylmorphinan-6-one hydrochloride (1.0 g, 2.73 mmol), prepared according to the described procedure^48^, in 3.5 ml of 48% HBr was refluxed for 15 min. After cooling, the brownish solution was evaporated, the residue treated with MeOH and again evaporated. The oily residue was crystallized from MeOH to yield 713 mg (66%) of colorless **6** ∙ **HBr**. Mp> 230 °C. (dec.). IR (KBr): 3545 and 3495 (^+^NH, OH), 1720 (CO) cm^−1^. ^1^H-NMR (DMSO-d_6_): δ 9.37 (s, OH), 8.65 (broad s, ^+^NH_2_), 6.64 (dd, J = 8.2, 8.2 Hz, 2 arom. H), 3.36 (s, *CH*_3_O), 1.48 (s, CH_3_-C(5)). MS (ESI) *m/z* 316 [M + 1]^+^. Anal. (C_18_H_21_NO_4_ ∙ HBr∙MeOH) C, H, N.

### Pharmacology. *drugs and chemical****s***

Cell culture media and supplements were obtained from Sigma-Aldrich Chemicals (St. Louis, MO), or Life Technologies (Carlsbad, CA). Radioligands [^3^H][D-Ala^2^,*N*-Me-Phe^4^,Gly-ol^5^]enkephalin ([^3^H]DAMGO, 50 Ci/mmol), [^3^H]Diprenorphine (37 Ci/mmol), and [^35^S]GTPγS (1250 Ci/mmol) were purchased from PerkinElmer (Boston, MA). [^3^H]HS665 (30.65 Ci/mmol) was prepared by Dr. Geza Toth (Institute of Biochemistry, Biological Research Centre, Hungarian Academy of Sciences, Szeged, Hungary) as previously described^49^. DAMGO, [D-Pen^2^,D-Pen^5^]enkephalin (DPDPE), U69,593, Diprenorphine, Tris(hydroxymethyl) aminomethane (Tris), 2-[4-(2-hydroxyethyl)piperazin-1-yl]ethanesulfonic acid (HEPES), Hank’s Balanced Salt Solution (HBSS), unlabeled GTPγS, guanosine diphosphate (GDP) and forskolin were obtained from Sigma-Aldrich Chemicals (St. Louis, MO). Morphine hydrochloride was obtained from Gatt-Koller GmbH (Innsbruck, Austria). Compounds **1a** and **2a** were synthesized according to the described procedure^13^, and 14-OMO (**3**) and 14-MM (**4**) were prepared as earlier described^16,50^. All other chemicals were of analytical grade and obtained from standard commercial sources.

### *In Vitro* Assays

#### Cell cultures

CHO cells stably expressing the human opioid receptors, µOR, δOR or κOR (CHO-hµOR, CHO-hδOR and CHO-hκOR cell lines), were kindly provided by Dr. Lawrence Toll (SRI International, Menlo Park, CA). The CHO-hµOR and CHO-hδOR cell lines were maintained in Dulbecco’s Minimal Essential Medium (DMEM)/Ham’s F-12 medium supplemented with fetal bovine serum (FBS, 10%), Penicillin/Streptomycin (0.1%), L-Glutamine (2 mM) and Geneticin (400 µg/ml). The CHO-hκOR cell line was maintained in DMEM supplemented with FBS (10%), Penicillin/Streptomycin (0.1%), L-Glutamine (2 mM) and Geneticin (400 µg/ml). CHO-hµOR or CHO-hδOR cells were stably transfected with the cAMP biosensor GloSensor-22F (Promega, Madison, WI), according to the previously described protocol^21^. Transfection was performed using the Viafect Transfection Reagent (Promega), according to the manufacturer’s instructions, and positive clones were selected with Hygromycin B (400 μg/mL). All cell cultures were maintained at 37 °C in 5% CO_2_ humidified air.

#### Competition binding assays

*In vitro* binding assays were conducted on human opioid receptors stably transfected into CHO cells according to the published procedures^20^. Briefly, CHO-hµOR, CHO-hδOR and CHO-hκOR cells grown at confluence were removed from the culture plates by scraping, homogenized in 50 mM Tris-HCl buffer (pH 7.4), using a Polytron homogenizer, then centrifuged once and washed by an additional centrifugation at 27,000 × *g* for 15 min, at 4 °C. The final pellet was resuspended in 50 mM Tris-HCl buffer (pH 7.4), and cell membranes (15–20 µg) were incubated with various concentrations of test compound and the appropriate radioligand [^3^H]DAMGO or [^3^H]Diprenorphine for 60 min at 25 °C, or [^3^H]HS665 for 30 min at 0 °C. Non-specific binding was determined using 1–10 µM of the unlabeled counterpart of each radioligand. Reactions were terminated by rapid filtration through Whatman glass GF/C fiber filters. Filters were washed three times with 5 mL of ice-cold 50 mM Tris-HCl buffer (pH 7.4) using a Brandel M24R cell harvester (Gaithersburg, MD). Radioactivity retained on the filters was counted by liquid scintillation counting using a Beckman Coulter LS6500 (Beckman Coulter Inc., Fullerton, CA). All experiments were performed in duplicate, and repeated at least three times with independently prepared samples.

[^35^*S]GTPγS Functional Assays*. Binding of [^35^S]GTPγS to membranes from CHO stably expressing the human opioid receptors was conducted according to the published procedures^13,20^. Cell membranes were prepared in Buffer A (20 mM HEPES, 10 mM MgCl_2_ and 100 mM NaCl, pH 7.4) as described for competitive radioligand binding assays. Cell membranes (5–10 µg) in Buffer A were incubated with 0.05 nM [^35^S]GTPγS, 10 µM GDP and various concentrations of test peptides in a final volume of 1 mL, for 60 min at 25 °C. Non-specific binding was determined using 10 µM GTPγS, and the basal binding was determined in the absence of test ligand. Samples were filtered over Whatman glass GF/B fiber filters and counted as described for competitive binding assays. All experiments were performed in duplicate, and repeated at least three times with independently prepared samples.

#### *cAMP accumulation assay*

Inhibition of the forskolin-stimulated intracellular cAMP accumulation in CHO cells co-expressing the hµOR and the cAMP biosensor GloSensor-22F (CHO-hµOR-p22F cells) and CHO cells co-expressing the hδOR and the cAMP biosensor GloSensor-22F (CHO-hδOR-p22F cells) was performed using the Glo-Sensor cAMP assay (Promega) according to the published procedure^21^. Cells were seeded in growth medium into 384-well plates at a density of 5,000 cells in 30 μL per well and incubated overnight. On the day of assay, culture media was removed, and cells were pre-equilibrated for 90 min with 4% v/v of the GloSensor cAMP reagent in reaction medium (20 mM HEPES, 1 x HBSS, pH 7.4) at 37 °C and 5% CO_2_. Cells were then treated with various concentrations of test compounds for 15 min at room temperature. Forskolin (10 μM) was added to each well, and luminescence was measured after 20 min using PerkinElmer Wallac Victor 1420 Mulitlable Counter. All experiments were performed in triplicate, and repeated at least three times with independently prepared samples.

### *In Vivo* assays

*Animals and Drug Administration*. *In vivo* studies were performed as described previously^20^. Male CD1 mice (30–35 g, 7–8 weeks old) were obtained from the Center of Biomodels and Experimental Medicine (CBEM) (Innsbruck, Austria). Mice were group-housed in a temperature controlled room with a 12 h light/dark cycle and with free access to food and water. All animal studies were conducted in accordance with ethical guidelines and animal welfare standards according to Austrian regulations for animal research, and were approved by the Committee of Animal Care of the Austrian Federal Ministry of Science and Research. Test compounds or vehicle (saline) were administered by s.c. route in a volume of 10 µL/1 g of body weight. Each experimental group included five to six animals. Separate groups of mice received the respective dose of compound, and individual mice were only used once for behavioral testing.

#### Tail-flick assay

The radiant heat tail-flick test was used to assess antinociceptive effects of test compounds after s.c. administration in mice, according to the original procedure of D’Amour and Smith^51^. The tail-flick test was performed using an UB 37360 Ugo Basile analgesiometer (Ugo Basile s.r.l., Varese, Italy. The reaction time required by the mouse to remove its tail after application of the radiant heat was measured and defined as the tail-flick latency (in seconds). Tail-flick latencies were measured before (basal latency, BL) and after drug or saline (control) s.c. administration (i.e. 30, 60 and 120 min) and (test latency, TL). A cut-off time of 10 s was used in order to minimize tissue damage.

#### Rotarod assay

Possible motor dysfunction or sedative effects of test compounds were assessed in mice using the rotarod test, as earlier described^52,53^. The accelerating rotarod treadmill (Acceler Rota-Rod 7650, Ugo Basile s.r.l., Varese, Italy) for mice (diameter 3.5 cm) was used. Animals were habituated to the equipment in two training sessions (30 min apart) one day before testing. On the experimental day, mice were placed on the rotarod, and treadmill was accelerated from 4 to 40 rpm over a period of 5 min. The time spent on the drum was recorded for each mouse before (baseline) and at 30 min after s.c. administration of saline (control) or test compound. Decreased latencies to fall in the rotarod test indicate impaired motor performance. A 300 s cut-off time was used.

### Data analysis

Data were analysed and graphically processed using GraphPad Prism 5.0. software (GraphPad Prism Software Inc., San Diego, CA, USA) and are presented as mean ± SEM. The K_i_ (nM), potency EC_50_ (nM), and efficacy E_max_ (%) values were determined from concentration-response curves by nonlinear regression analysis. The K_i_ values were determined by the method of Cheng and Prusoff^54^. In the [^35^S]GTPγS binding assays, efficacy was determined relative to the reference full opioid agonists, DAMGO (µOR), DPDPE (δOR), and U69,593 (κOR). In the cAMP accumulation assay, efficacy was determined relative to the reference µOR agonist DAMGO. The antinociceptive effect (as percentage of Maximum Possible Effect, %MPE) was calculated according to the formula = [(TL – BL)/(cut-off time – BL)] × 100, and the dose necessary to produce a 50% MPE (ED_50_) and 95% confidence limits (95% CL) were determined using the method of Litchfield and Wilcoxon^55^. In the rotarod test, percentage (%) changes from the rotarod latencies obtained before (baseline, B) and after drug administration (test, T) were calculated as: 100 × (T/B). Data were statistically evaluated using unpaired t-test, one-way ANOVA with Tukey’s multiple comparison *post hoc* test, or two-way ANOVA with significance set at *P* < 0.05.

### Molecular modeling

The structure of the human µOR was remodeled based on the crystal structure of the murine µOR (PDB ID: 5C1M)^38^ by using the mutation tool of MOE (Molecular Operating Environment), 2014.09; Chemical Computing Group Inc.) with subsequent sidechain optimization. We used the active crystal structures of the δOR (PDB ID: 6PT3)^45^ and κOR (PDB ID: 6B73)^46^ for docking experiments. All receptor-ligand docking experiments were performed with the CCDCs software GOLD version 5.1^56^. Water molecules and ligands were removed and correct protonation states were assigned. All residues of the inner receptor core region and the C-terminal domain were defined as potential binding site (12 Å around the γ-carbon atom of D147; PDB ID: 5C1M). For receptor-ligand docking default settings were applied and GoldScore served as scoring function. All obtained docking poses and receptor-ligand interactions were analyzed using LigandScout 4.2^40^ using a 3D-pharmacophore approach. All-atoms MD simulations were performed in triplicates with Desmond version 2018-3 on the Curta compute cluster of the Freie Universität Berlin. All conditions and settings used for system building and simulation were chosen based on a previously reported protocol^47^.

## Supplementary Information

Supporting Information.
