# Supplementary material for: N-Phenethyl Substitution in 14-Methoxy-N-methylmorphinan-6-ones Turns Selective µ Opioid Receptor Ligands into Dual µ/δ Opioid Receptor Agonists
Source: Sci Rep. 2020 Mar 27;10:5653. doi: 10.1038/s41598-020-62530-w (PMC7101422; doi:10.1038/s41598-020-62530-w)
Supplement: Supplementary file 1 — Supporting Information. [file 41598_2020_62530_MOESM1_ESM.pdf]

Supplementary Information

N-Phenethyl Substitution in 14-Methoxy-*N*-methylmorphinan-6-ones Turns Selective μ Opioid Receptor Ligands into Dual μ/δ Opioid Receptor Agonists

Maria Dumitrascuta,[1§] Marcel Bermudez,[2§] Tanila Ben Haddou,[1] Elena Guerrieri,[1] Lea Schläfer,[1] Andreas Ritsch,[1] Sandor Hosztafi,[3] Aquilino Lanterro,[1] Christoph Kreutz,[4] Dominique Massotte,[5] Helmut Schmidhammer,[1] Gerhard Wolber,[2] Mariana Spetea[1]*

[1]Department of Pharmaceutical Chemistry, Institute of Pharmacy and Center for Molecular Biosciences Innsbruck (CMBI), University of Innsbruck, 6020 Innsbruck, Austria
[2]Institute of Pharmacy, Freie Universität Berlin, D-14195 Berlin, Germany
[3]Department of Pharmaceutical Chemistry, Semmelweis University, H-1092, Budapest, Hungary
[4]Institute of Organic Chemistry, Center for Molecular Biosciences Innsbruck (CMBI), University of Innsbruck, 6020 Innsbruck, Austria
[5]Centre de la Recherche Nationale Scientifique, Université de Strasbourg, Institut des Neurosciences Cellulaires et Intégratives, 67000 Strasbourg, France

*Correspondence to:
Mariana Spetea
E-mail: Mariana.Spetea@uibk.ac.at
Phone: +43-512 507 58277

[§]These authors contributed equally.

Table of Contents

S2: Figure S1
S3: Figure S2
S4: Figure S3
S5: Figure S4
S6: Figure S5

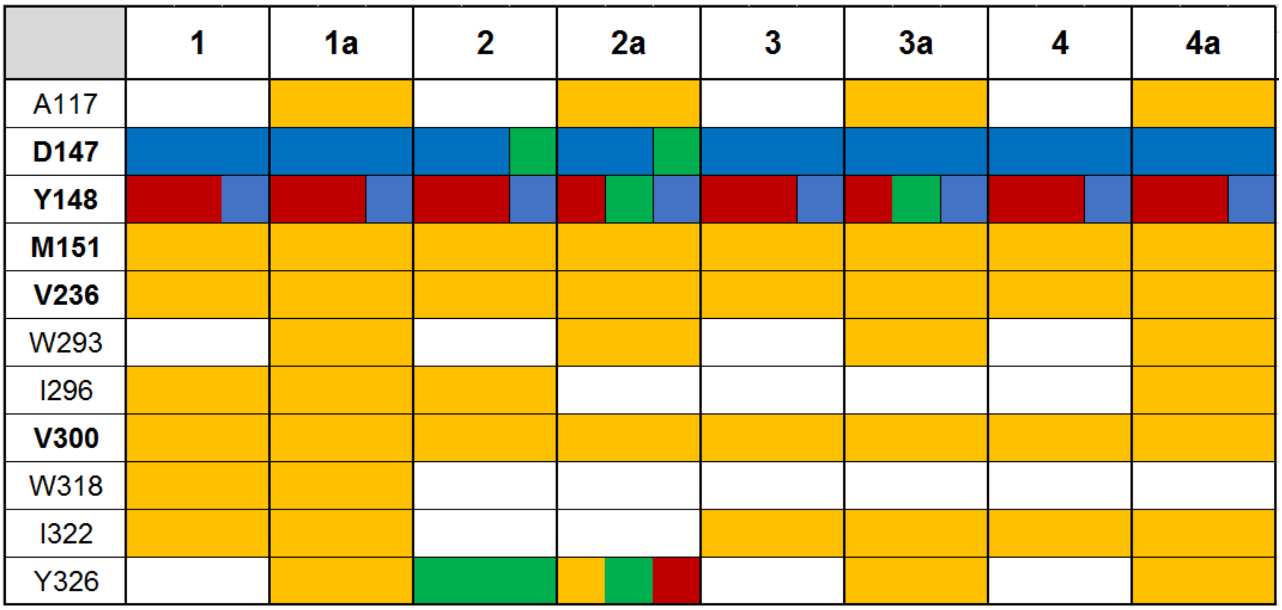

Figure S1. Ligand-µOR interaction patterns derived from molecular docking solutions of *N*-methylmorphinans **1-4** and their respective *N*-phenethyl analogues **1a-4a**. Yellow fields indicate lipophilic contacts, red fields represent hydrogen bond acceptors, green fields represent hydrogen bond donors and positively charged centers are shown as blue fields. White fields indicate the absence of an interaction with that residue. Residues which show the same type of interaction for all morphinan ligands are marked in bold.

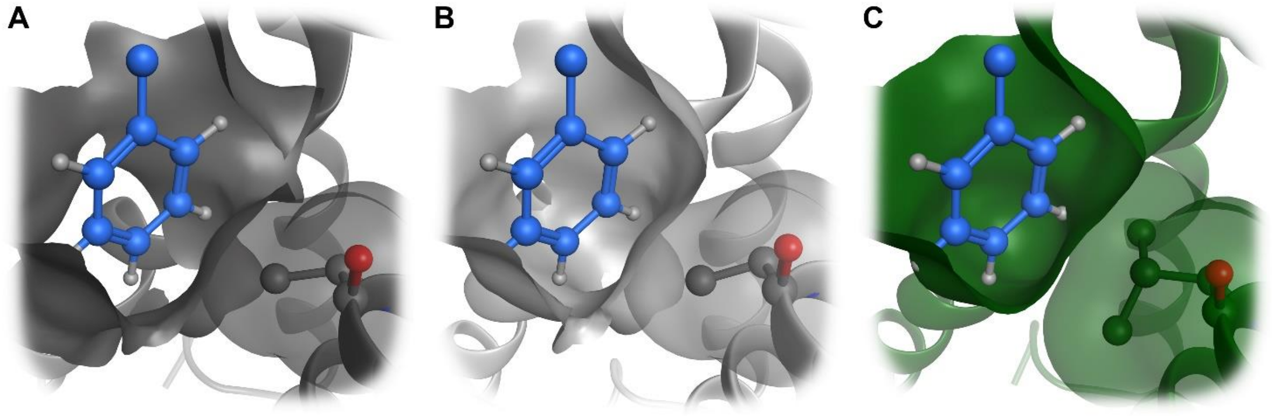

Figure S2. The phenyl ring system (blue) is embedded in a lipophilic subpocket. While the µOR (A) and the δOR (B) have an alanine residue at position 2.53, the κOR has a valine at this position. This reduces the size of the lipophilic subpocket in the κOR and represent a steric hindrance to optimally host a phenyl ring.

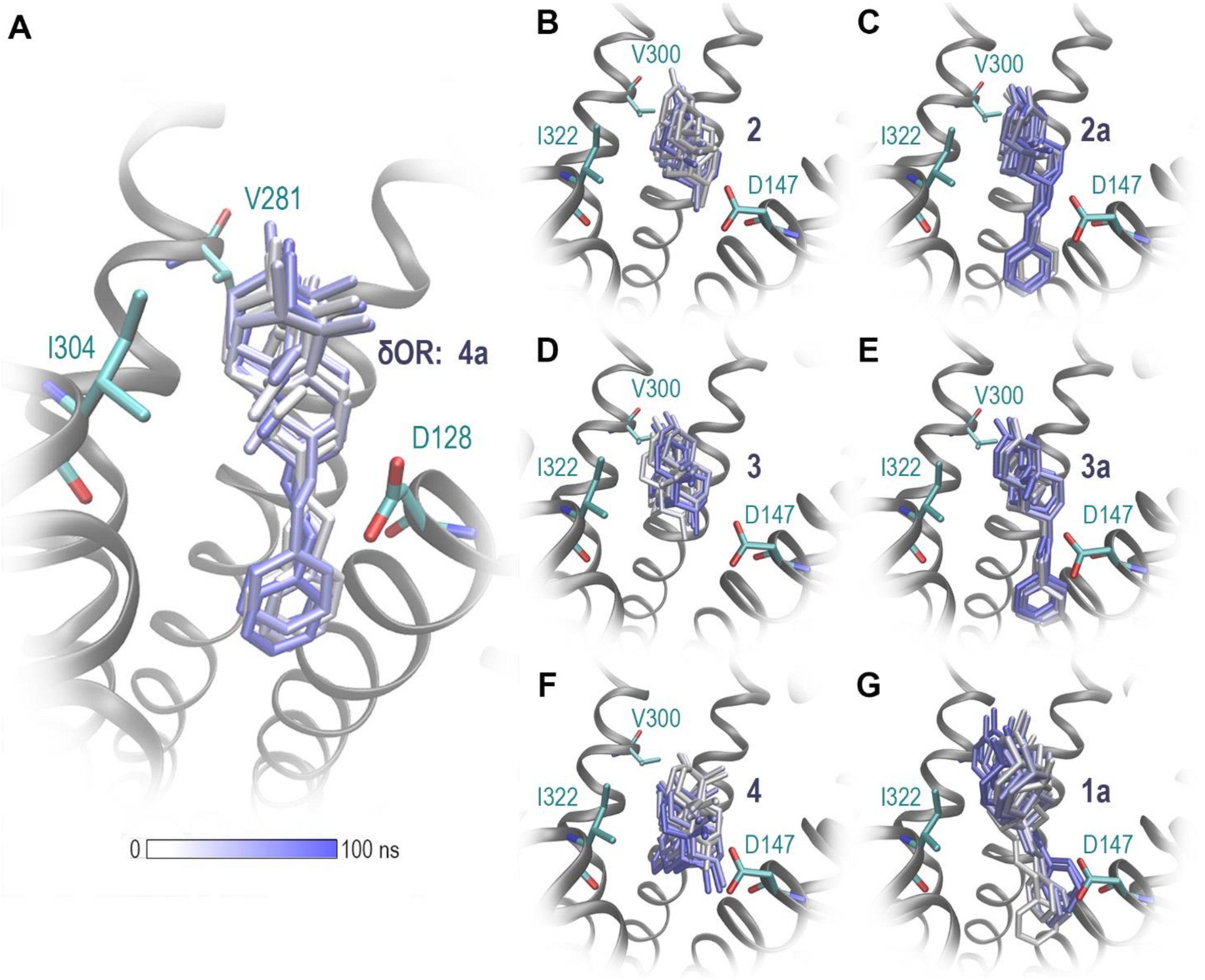

Figure S3. All-atoms MD simulations support the docking results. The binding mode of **4a** in the structural δOR model (A) is comparable to the µOR (see Figure 4 in the main text). The results are in accordance with the δOR crystal structure (PDB ID: 6PT3) The binding location and the major ligand orientation in the binding pocket for compounds **1a**, **2**, **2a**, **3**, **3a**, and **4** in complex with the µOR (PDB ID: 5C1M) are visualized in panels B-G. All key interactions reported from the docking experiments remain firm over 100 ns of MD simulations for all investigated compounds.

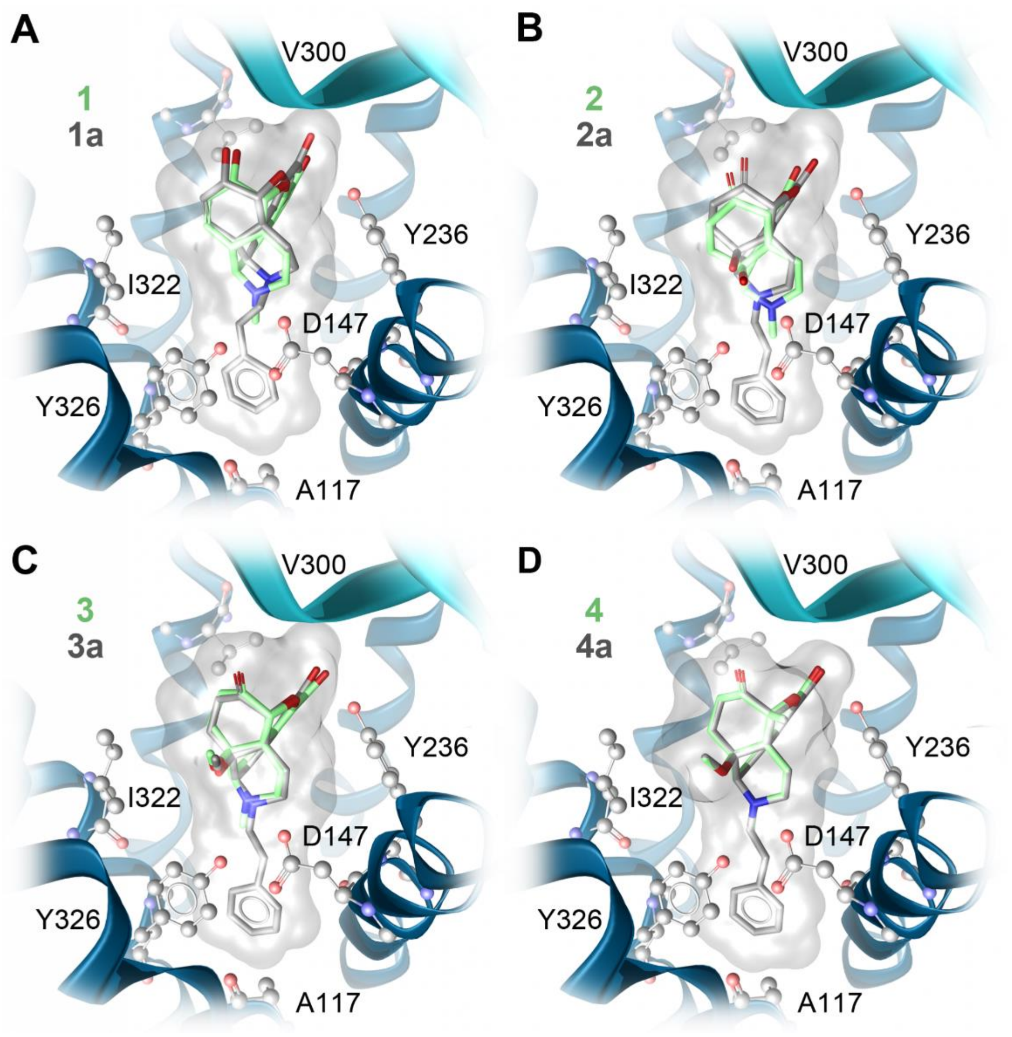

Figure S4. Docking of *N*-methylmorphinans **1**-**4** (green) and their respective *N*-phenethyl analogues **1a**-**4a** (gray) to the active structure of μOR (PDB ID: 5C1M). (A) Overlay of morphine (**1**) and **1a**. (B) Overlay of oxymorphone (**2**) and **2a**. (C) Overlay of 14-OMO (**3**) and **3a**. (D) Overlay of 14-MM (**4**) and **4a**.

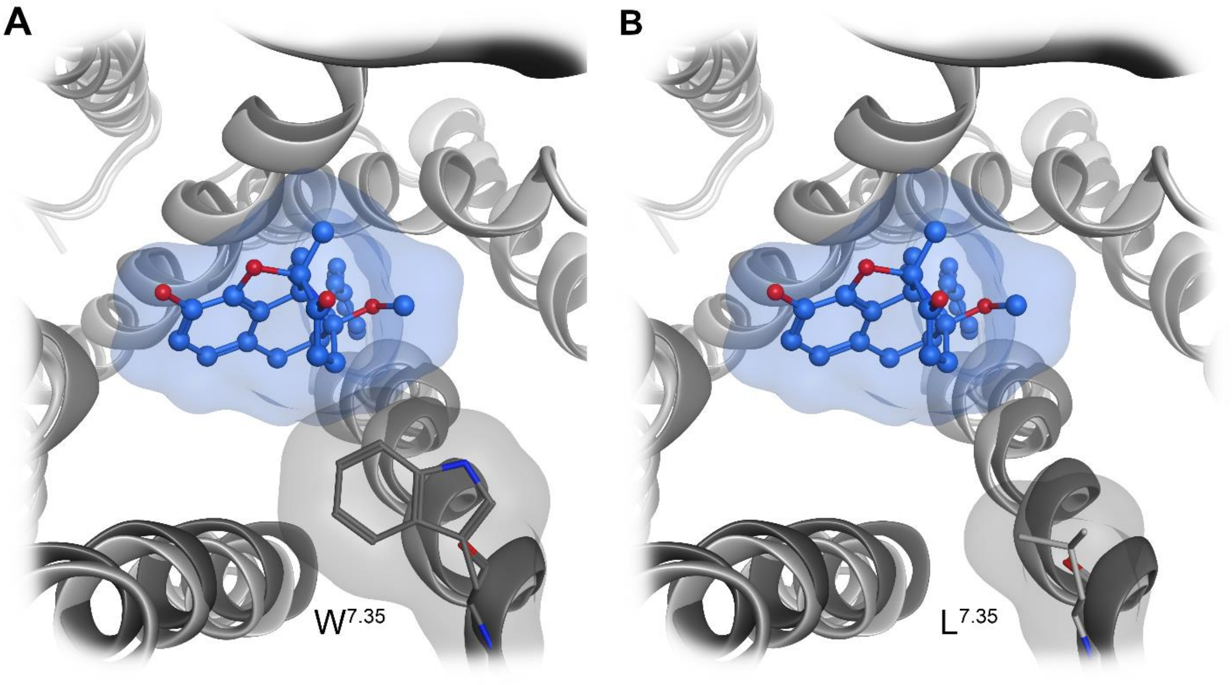

Figure S5. A comparison of the μOR and the δOR indicates a major difference at the beginning of helix seven at position 7.35. Whereas the tryptophan of the μOR (A) can form lipophilic contacts with the morphinan ring system of **4a** (blue), the leucine residue of the δOR cannot directly contribute to ligand binding (B).
